# Preserving Function, Enhancing Precision: The Rise of Personalized Endonasal Sinus Surgery

**DOI:** 10.7759/cureus.100036

**Published:** 2025-12-24

**Authors:** Lino Di Rienzo Businco, Abdillah Hasbi Assadyk

**Affiliations:** 1 Department of Otorhinolaryngology, UBRI (Ultrastructural Biophysics Research Institute) Institute and San Giovanni Battista Hospital, Order of Malta, Rome, ITA; 2 Department of Ear, Nose, and Throat, Mandaya Royal Hospital Puri, Tangerang, IDN

**Keywords:** balloon sinuplasty, chronic rhinosinusitis, endoscopic sinus surgery, kinetic oscillation stimulation, mucosal preservation, personalized surgery, quantum molecular resonance, thinvasive

## Abstract

Chronic rhinosinusitis (CRS) is a common disease with increasing global prevalence that significantly impacts patients' quality of life. Advances in surgical technology and a better understanding of mucosal pathophysiology have transformed its management landscape. However, a notable gap remains in the published literature. This article reviews the evolution of endonasal sinus surgery and presents a position on the emerging concept of “Thinvasive” surgery, a minimally invasive and function-preserving paradigm for the modern management of CRS.

A comprehensive review of the historical development, current techniques, and novel approaches in endoscopic sinus surgery was conducted. The transition from traditional external procedures, such as Caldwell-Luc, to functional endoscopic sinus surgery (FESS) marked a pivotal shift toward mucosal preservation and restoration of physiological drainage. Further advancements, such as image-guided FESS, balloon sinuplasty, and other minimal-access modalities, have facilitated more precise and individualized interventions. The Thinvasive approach integrates these innovations with patient-specific assessment strategies, including radiologic and functional evaluation tools like the Sino-Nasal Outcome Test-22, quantum molecular resonance (Telea Medical, Sandrigo, Italy), and Ozilia-kinetic oscillation stimulation (Chordate Medical, Kista, Sweden).

The future of endonasal sinus surgery lies in a flexible and personalized continuum. Thinvasive techniques offer a promising hybrid between medical and surgical treatment. Nonetheless, further research is needed to establish their long-term efficacy and to develop standardized patient selection frameworks.

## Introduction and background

Chronic rhinosinusitis (CRS) is a prevalent otolaryngologic disease, characterized by a chronic inflammation of the mucosa in the nasal cavity and paranasal sinuses that persists for at least 12 weeks [1]. In recent decades, the prevalence of CRS has increased nearly fourfold, affecting approximately 8.7% of adults worldwide, with higher rates seen in Europe and North America [2-4].

Nasal endoscopy has emerged as a crucial instrument in the diagnosis and management of CRS in modern clinical practice [5]. This method enables direct visualization of the nasal cavity and sinus drainage pathways, providing objective evidence of mucosal inflammation, polypoid formation, or anatomical abnormalities [5]. In surgical planning, endoscopy also aids in identifying the extent of disease and tailoring the approach, particularly in refractory cases, where endoscopic sinus surgery is considered the gold standard [6].

While endoscopic examination of the sinonasal cavity originated early in 1901, endoscopic sinus surgery was not routinely performed until the 1970s [6,7]. Prior to its introduction, sinus surgery typically involved invasive external procedures that were destructive to the mucosa and primarily aimed at removing diseased tissue. One of the most recognized methods was the Caldwell-Luc, an open maxillary sinus surgery that was later associated with considerable complications [8,9]. A paradigm shift occurred with the introduction of functional endoscopic sinus surgery (FESS), a minimally invasive surgical technique that uses a nasal endoscope to target the osteomeatal complex for restoring natural sinus drainage and preserving healthy mucosa [7].

Since its inception, the techniques employed in endoscopic surgery have continuously evolved, driven by technological innovations, advances in surgical instruments, and enhanced imaging modalities. Contemporary FESS techniques now incorporate precision tools and image-guidance systems, facilitating personalized surgical interventions tailored to the individual anatomy and pathology of patients. Innovations such as image-guided FESS [10], balloon sinuplasty [11], and minimally invasive "Thinvasive" procedures [12] exemplify the ongoing refinement and sophistication of endoscopic sinus surgery.

This article aims to study the evolution of endonasal sinus surgery, tracing its development from traditional approaches to modern, personalized methodologies. It also presents as a position paper on the emerging concept of Thinvasive surgery, emphasizing minimally invasive and maximally conservative, function-preserving strategies in the management of CRS, and finalized to intercept and modify the natural evolution from rhinitis to rhinosinusitis in an early stage.

## Review

Methodology

This manuscript was designed as a narrative review and position paper focusing on the historical evolution, current practices, and emerging concepts in endonasal sinus surgery, with particular emphasis on function-preserving and personalized surgical strategies in the management of CRS.

A targeted literature search was conducted using PubMed, Scopus, and Google Scholar to identify relevant publications. The primary search terms included combinations of chronic rhinosinusitis, endoscopic sinus surgery, functional endoscopic sinus surgery, balloon sinuplasty, minimally invasive sinus surgery, mucosal preservation, thinvasive surgery, quantum molecular resonance, and kinetic oscillation stimulation. Additional articles were identified through manual screening of reference lists from key publications.

The literature selection prioritized studies that contributed to the understanding of the conceptual development, technical evolution, clinical application, and functional outcomes of endonasal sinus surgery. Both landmark historical articles and contemporary clinical studies were included to provide a comprehensive perspective. Given the narrative and conceptual nature of this review, no formal inclusion or exclusion criteria based on study design were applied, and no quantitative synthesis or meta-analysis was performed.

The selection of articles was guided by their relevance to surgical philosophy, technological innovation, and function-preserving approaches, rather than by predefined methodological quality thresholds. Consequently, a formal risk-of-bias assessment was not undertaken, as the purpose of this manuscript was to synthesize existing knowledge and propose a conceptual framework rather than to evaluate intervention efficacy quantitatively.

The final synthesis integrates historical context, technological advancements, and clinical considerations to support a personalized, continuum-based approach to CRS management, culminating in the proposed “Thinvasive” paradigm. This approach reflects current trends toward precision medicine and individualized surgical decision-making in 

Study Selection

Studies were considered for inclusion if they met one or more of the following criteria: (1) provided historical, anatomical, or conceptual insights into the development of sinus surgery techniques; (2) described surgical principles, technical innovations, or clinical applications of endonasal sinus surgery; (4) addressed functional outcomes, mucosal preservation, or personalized approaches in the management of chronic rhinosinusitis; (5) included landmark publications, consensus statements, randomized trials, observational studies, or relevant narrative reviews that contributed meaningfully to the evolution of the field. Both historical landmark studies and contemporary clinical literature were intentionally included to illustrate the progression of surgical philosophy and technology over time.

Publications were excluded if they were: (1) unrelated to endonasal or endoscopic approaches to sinus surgery; (2) focused exclusively on non-surgical management without relevance to surgical decision-making; (3) were single case reports with no conceptual, technical, or educational contribution to the evolution of sinus surgery; (4) lacked sufficient methodological description or clinical relevance to support their inclusion in a conceptual synthesis.

Study Appraisal and Synthesis

Given the narrative and position-based nature of this review, formal quality scoring, risk-of-bias assessment, or quantitative synthesis was not performed. Articles were selected based on their relevance, clinical significance, and contribution to surgical philosophy and innovation, rather than predefined methodological thresholds. The final synthesis was developed through thematic integration of historical context, technological advancements, and contemporary clinical perspectives to support a function-preserving, personalized continuum of care in chronic rhinosinusitis.

Historical and technical landmarks

Pre-endoscopic Era: External Approaches and Their Limitations

The earliest documented recognition of sinus anatomy dates back to 1651, when English physician Nathaniel Highmore described the maxillary sinus as the Antrum Highmore [13]. Subsequently, early concepts of sinus disease were rooted in the idea of persistent infection and pus formation as the primary etiology, guiding surgical strategies toward mechanical drainage and debridement [14]. Introduced in the late nineteenth century, the Caldwell-Luc procedure became the most widely adopted technique during the pre-endoscopic era [15]. The procedure involved creating a bony opening through the anterior wall of the maxillary sinus via the canine fossa to facilitate drainage and removal of infected tissue. Although effective in providing temporary symptom relief, it was later associated with long-term complications, including infraorbital nerve injury, fistula formation, dacryocystitis, and disruption of normal sinus mucociliary function, often resulting in recurrent polyps and sinusitis [8,14].

Rooted in similar surgical principles, external procedures were also developed to address disease of the ethmoid and frontal sinuses. Techniques such as external ethmoidectomy, typically performed via a Lynch incision along the medial orbital rim, and frontal sinus trephination, which involved creating an access point above the eyebrow, allowed direct tissue debridement [8,16]. However, these external approaches offered limited visualization of the sinus mucosa and carried substantial risks, including facial skin scarring, orbital trauma, and cerebrospinal fluid leaks [8,17].

Emergence of Functional Endoscopic Sinus Surgery

Before the advent of endoscopic techniques, surgical approaches to chronic sinus disease primarily targeted larger sinuses, such as the maxillary and frontal sinuses, due to their prominent clinical and radiographic manifestations [18]. The introduction of FESS in the mid-1980s revolutionized both the surgical technique and the pathophysiological understanding of CRS [7,18].

The paradigm shift was primarily influenced by the pioneering work of Walter Messerklinger in the 1960s [18]. Using an endoscopic examination of fresh cadavers, this study describes the anatomy of the paranasal sinuses and the physiological role of mucociliary clearance, with a particular attention to the osteomeatal complex (OMC) [7]. The study revealed that chronic sinus disease originated in the ethmoid region due to anatomical variations within the OMC that impaired ventilation and clearance, leading to secondary involvement of larger sinuses [7]. Although contemporary perspectives now recognize CRS as a multifactorial disease involving immune, microbial, and environmental factors, Messerklinger's focus on the rhinogenic origin of disease provided the initial pathophysiological framework [18].

The widespread availability of nasal endoscopy and computed tomography (CT) imaging by the late 1980s further enabled objective diagnosis and guided intervention for chronic sinus disease [19]. In 1985, Kennedy and colleagues formally introduced the term FESS, demonstrating the effectiveness of rigid fibreoptic endoscopes in precisely treating disease areas while preserving healthy mucosa [20]. The surgical approach emphasized targeted intervention within the ethmoid sinuses, widening of narrowed drainage pathways, and clearance of inflammation from the prechambers leading to the frontal and maxillary sinuses [21]. Those early limited procedures led to the current standardized surgical protocols of FESS, comprising ethmoidectomy, uncinectomy, and middle meatal antrostomy, designed to restore sinus ventilation and drainage through the natural physiological route.

Integration of Technology in FESS: Image-Guidance and Surgical Instrument

An initial advancement of sinonasal endoscopic visualization occurred with the development of the rod-lens system by Harold Hopkins in 1950 [7]. This innovation laid the foundation for modern rigid endoscopes, which possess the ability to visualize narrow sinus cavities with more clarity. Following the introduction of FESS, nasal endoscopy was subsequently combined with routine high-resolution CT imaging to form an integrated diagnostic framework, providing detailed anatomical information beneficial for surgical planning [22]. Introduced in the late 1990s, image-guided surgery utilizing computer-assisted navigation systems further enhanced localization accuracy in FESS, proving valuable in anatomically complex regions, such as the frontal recess, sphenoethmoidal area, and skull base, as well as in revision surgeries or cases with distorted anatomy due to extensive inflammation [23,24]. Further progress came with the development of real-time three-dimensional navigation systems, which offered improved accuracy in identifying the spatial relationships between surgical instruments and anatomical landmarks [25]. These advanced systems, often integrated with CT/magnetic resonance imaging fusion, are now established and used in complicated cases, revisions, and skull base extension surgery [19].

In parallel with the evolution of visualization technology, the cutting instrumentation used in sinus surgery also underwent significant refinement. Early procedures relied on manually operated instruments specifically designed for intranasal use, such as Blakesley forceps, through-cutting forceps, and sickle knives [26]. While these tools allowed for targeted tissue removal within the sinonasal cavity, they were limited by reduced precision, slower operative speed, and increased risk when working in anatomically complex regions. In the 1990s, the introduction of a powered device called the microdebrider marked an important advancement in FESS [7]. Originally designed for orthopaedic surgery, the instrument features an oscillating blade within a suction cannula, allowing for simultaneous tissue resection and suction [27]. The device continues to be utilized in contemporary ear, nose, and throat practice with enhancements, including the development of sharper blades, various angulated designs, and a range of diameters to suit specific anatomical challenges. These innovations enabled more controlled and efficient tissue removal, minimized mucosal trauma, and reduced operative time. One of the limitations of using a microdebrider in FESS is the lack of tactile feedback, which, when combined with potential intraoperative disorientation, may increase the risk of orbital or skull base injury. However, recent studies have demonstrated that device failures and associated patient injury are infrequent occurrences [28,29].

Mainstreaming of Balloon Sinuplasty: Toward Minimally Invasive Alternatives

**Figure 1:**
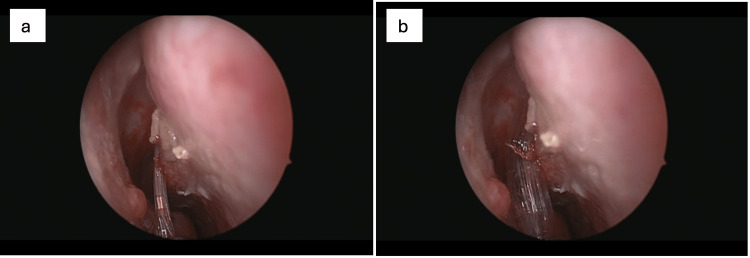
Endoscopic view of the right nasal cavity demonstrating balloon catheter placement within the right middle meatus prior to inflation (a). The anatomical landmarks, including the middle turbinate and adjacent mucosa, remain undistorted, indicating correct positioning of the balloon catheter at the target site for OMC dilation. Initial inflation of the balloon catheter within the right middle meatus (b). The balloon is expanding the anatomical space, displacing adjacent structures to allow remodeling of the OMC. Note the blanching of the surrounding mucosa, indicating successful tissue distension. Consent from the patient to submit images has been obtained prior to submission to the journal.

The introduction of balloon sinuplasty (BSP) in the early 2000s marked a pivotal advancement in the evolution of minimally invasive endonasal methodologies for the management of CRS. Unlike conventional FESS, BSP was developed as a catheter-based procedure that achieves sinus ostial dilation without the need for bone or tissue removal (Figure 1). This approach aimed to preserve mucosal integrity and reduce surgical morbidity.

Early feasibility was demonstrated in a cadaveric study by Bolger and Vaughan [30], who successfully applied the balloon dilation technique to the maxillary, sphenoid, and frontal ostia or recesses. Their study revealed that the procedure resulted in minimal mucosal damage and decreased risk to adjacent structures as confirmed through CT imaging, endoscopic evaluation, and gross anatomical dissection [30].

The initial large-scale clinical evaluation came from a multicenter registry involving 1,036 patients with uncomplicated CRS [11]. The study reported favorable outcomes, including no major adverse events, a low revision rate, and a significantly reduced incidence and severity of postoperative sinus infections as compared to FESS outcomes [11]. Additionally, patients who underwent standalone BSP were associated with reduced intraoperative blood loss, shorter operative time, and fewer post-operative debridements compared to those who received concomitant ethmoidectomy [11,31].

Subsequent randomized controlled trials (RCTs) provided more evidence supporting the clinical effectiveness of BSP [32]. In 2011, Plaza et al demonstrated that balloon dilation of the frontal recess significantly improved obstruction, as measured by the CT-scan finding of Lund-Mackay stage, with results comparable to those achieved through a Draf 1 endoscopic procedure [32]. A separate multicenter RCT conducted in 2014 assessed one-year outcomes in adults with maxillary CRS and showed that BSP was equally effective as FESS, both in patients with isolated maxillary disease and in those with limited anterior ethmoid involvement [33]. The study reported notable improvements in quality of life, ostial patency, and frequency of symptom recurrence [33]. In addition to the adult population, a prospective study by Liu et al. [34] also demonstrated that BSP was a safe and effective technique in treating paediatric patients with refractory CRS from medical therapy.

However, most RCTs and registry data have excluded patients with complex or severe sinonasal diseases such as those with extensive nasal polyposis, osteitis, or connective tissue disorder [35]. In such cases, the anatomical obstruction is often too advanced for a purely dilational technique. Recent clinical reviews emphasize that BSP should not be employed as a stand-alone therapy in these patients, who typically require more extensive surgical management through conventional FESS [36].

Thinvasive Surgery: A Position on Function-Preserving Strategies in Sinonasal Disease

The field of endoscopic sinus surgery continues to evolve, with increasing emphasis on preserving physiological function, minimizing iatrogenic trauma, and tailoring treatment to individual patient characteristics. Among recent developments is the concept of “Thinvasive” surgery, a term that merges the principles of minimal invasiveness with tissue-sparing techniques [12]. Initially developed by Professor Lino Di Rienzo Businco and colleagues, this approach integrates elements of conventional FESS with emerging technologies aimed at supporting mucosal preservation, modifying the key endonasal areas where the disease arises in a precise and personalized manner, and accelerating postoperative recovery [12].

**Figure 2:**
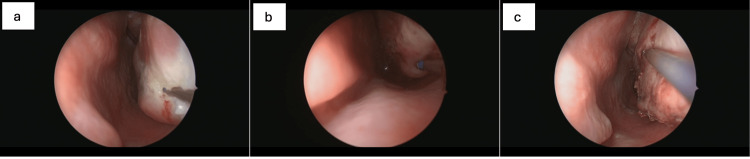
Endoscopic view of the left nasal cavity demonstrating the initiation of QMR energy application to the anterior-inferior segment of the inferior turbinate (a). The tip of the QMR probe is in contact with the edematous mucosa, targeting submucosal tissues for volume reduction while preserving the mucosal surface. Posterior view of the left inferior turbinate, where QMR therapy is applied to the caudal end of the turbinate (b). The image shows mucosal blanching and the beginning of submucosal tissue contraction, indicating effective energy delivery. The medial-superior area of the left inferior turbinate was treated using QMR, highlighting precision application to areas closer to the septum (c). The superior turbinate border remains intact, emphasizing tissue preservation. Consent from the patient to submit images has been obtained prior to submission to the journal.

Central to the Thinvasive methodology is the use of high-precision balloon dilation systems. Unlike earlier-generation balloon devices, which primarily focused on ostial patency, the newer systems utilized in this approach are designed to maintain the integrity of sinonasal architecture while enhancing mucociliary clearance. Improvements in balloon mechanics and inflation control enable targeted reshaping of sinus ostia with minimal disruption to adjacent bone and soft tissue [12]. An additional component of the approach involves the integration with adjunctive modalities such as quantum molecular resonance (QMR; Telea Medical, Sandrigo, Italy) and Ozilia-kinetic oscillation stimulation (Ozilia-KOS; Chordate Medical, Kista, Sweden). QMR utilizes non-thermal, oscillating energy to interact with cell membranes, promoting tissue repair and regeneration without thermal injury [37]. Applications in turbinate reduction (Figure 2), adenoid ablation, and post-balloon mucosal modulation have shown promise in enhancing healing and reducing the risk of recurrence. In a study by Ricciardiello et al., QMR demonstrated significant improvement in nasal airflow and symptom control in patients with inferior turbinate hypertrophy, with sustained benefits on follow-up [12,38].

Thinvasive protocols emphasize individualized treatment planning. Rather than following uniform surgical algorithms, decision-making is informed by detailed preoperative assessment, including endoscopic findings, radiologic imaging, and immunologic-allergologic profiling [12,39]. For instance, patients with mucosal hyperreactivity or comorbid allergic rhinitis may be more appropriate candidates for mucosal preservation strategies, such as QMR, whereas individuals with anatomically narrowed frontal recesses may benefit from selective balloon dilation. This approach aligns with evidence from randomized trials, such as that by Bikhazi et al., which demonstrated that balloon-only interventions can yield outcomes comparable to traditional FESS, with fewer complications and more rapid recovery [33].

Integrating medical therapy, functional stratification, and surgical decision-making

Role of Medical Therapy in a Surgical Landscape

Despite significant advancements in surgical techniques for CRS, accurate diagnosis and initiation of appropriate first-line medical therapy remain the cornerstone of effective management. Pharmacologic treatment using primarily intranasal corticosteroids and saline irrigation continues to be the preferred and evidence-based initial approach, particularly for patients with bilateral and diffuse disease. In cases where CRS is driven by underlying allergic sensitization, patients may derive additional benefit from allergen-specific immunotherapy, provided that clinically relevant allergens have been accurately identified through appropriate diagnostic testing [40]. However, patients with unilateral CRS often exhibit suboptimal responses to medical therapy and may benefit from earlier surgical intervention [41]. In such cases, non-contrast CT imaging and disease endotyping are essential to guide surgical decision-making.

In adults with uncomplicated CRS, Endoscopic Sinus Surgery(ESS) may be considered appropriate when the CT Lund-Mackay score is ≥1, following at least eight weeks of intranasal corticosteroids [42]. For CRS with nasal polyps, this should include a short course of systemic steroids. For CRS without nasal polyps, either a short course of antibiotics or prolonged low-dose anti-inflammatory antibiotics is recommended. Surgery is generally reserved for cases with a post-treatment Sinonasal Outcome Test-22 (SNOT-22) score of ≥20. These criteria aim to reduce unnecessary surgeries, which have been shown to result in minimal improvement in postoperative symptoms and quality of life [1,41-43].

Although current clinical guidelines emphasize optimizing medical therapy before considering surgery, the definition of “adequate medical therapy” and the optimal surgical timing remain subjects of ongoing debate [44]. The use of oral antibiotics and systemic steroids remains contentious due to insufficient supporting evidence and concern regarding the side effects [41]. Prior studies also suggest that delayed surgical intervention in appropriate CRS patients may result in diminished symptom improvement [45,46]. Although the evidence is still limited and controversial, with a prior study reporting no significant difference in SNOT-22 score improvement [47], patients undergoing earlier surgery are associated with reduced downstream healthcare utilization, including fewer clinic visits, medication prescriptions, and adjunctive therapies [46].

From a pathophysiology standpoint, persistent type 2 inflammation in CRS promotes mucosal remodelling such as fibrosis and basement membrane thickening [48]. These histopathologic alterations may become irreversible over time, potentially diminishing the efficacy of both medical and surgical therapies [48]. This underscores the importance of timely surgical intervention as part of a proactive management strategy aimed at halting disease progression, preserving mucosal function, and optimizing long-term clinical outcomes.

By leveraging medical therapy not only as a treatment but also as a diagnostic tool to identify recalcitrant patients, clinicians can better select candidates for surgery and tailor the extent of intervention. This approach reduces unnecessary surgical burden and aligns management with the patient's unique disease profile.

Functional Stratification: Role of Symptom Assessment and Ozilia-Kinetic Oscillation Stimulation

Although CT imaging remains essential for surgical planning, radiologic findings often do not reflect the severity of symptoms or the degree of functional impairment reported by patients. The Lund-Mackay staging system provides a standardized method for grading disease extent on CT, but prior studies have shown that its correlation with quality-of-life outcomes is limited [49,50]. Mucosal thickening or sinus opacification is often present even in patients without significant sinonasal symptoms [51].

Given these limitations, clinical decision-making should incorporate functional outcome measures alongside imaging. The SNOT-22 is a well-validated symptom score that helps quantify the subjective burden of disease. Studies suggest that higher preoperative SNOT-22 scores are associated with an increased likelihood of electing surgery and a higher chance of postoperative improvement [52,53]. Changes in SNOT-22 scores after surgery have also been proposed as indicators of treatment response and predictors of revision risk [54].

In this context, Ozilia-KOS has emerged as a non-invasive outpatient option that may contribute to both therapeutic relief and functional assessment. This technique delivers low-frequency oscillatory pressure via an intranasal catheter, aiming to enhance mucociliary transport and reduce congestion. Early data, including studies in patients with chronic rhinitis, suggest that Ozilia-KOS can improve subjective nasal symptoms, potentially reflecting underlying mucosal health [55-57].

When incorporated into a structured treatment pathway, Ozilia-KOS may help identify patients with preserved mucociliary function who are more likely to benefit from continued medical therapy. A positive response could indicate that surgery may be deferred in favor of conservative measures. This role is currently being explored in prospective trials, such as NCT03399721, which aim to define the utility of Ozilia-KOS in preoperative stratification of patients with CRS.

The use of Ozilia-KOS within a broader clinical framework is consistent with recommendations from EPOS 2020, which emphasize individualized treatment plans based on radiologic findings, symptom severity, and physiologic function [41]. In patients with ambiguous radiologic features or milder disease, Ozilia-KOS may offer an additional layer of information to support evidence-based decision-making and reduce the risk of unnecessary surgery.

Unified Algorithm: From Therapy to Tailored Surgery

The authors proposed an updated management framework of CRS that emphasizes a unified, patient-centered algorithm integrating optimized medical therapy, radiological and functional assessments, and selective surgical intervention. This approach departs from the traditional linear pathway and instead supports a cyclical, individualized evaluation based on disease severity, treatment response, and patient-specific factors (Table 1).

**Table 1:** Evolution of CRS management procedures from a different era and the proposition of a new algorithm CRS: chronic rhinosinusitis

Era / Procedure	Core Principle	Typical Surgical Approach	Strengths	Limitations
Pre-Endoscopic Era – Caldwell–Luc Procedure	Radical drainage and removal of diseased sinus mucosa	External maxillary sinus access via the canine fossa	Effective short-term symptom relief; wide exposure of the maxillary sinus	Highly invasive; mucosal destruction; disruption of mucociliary clearance; risk of infraorbital nerve injury, facial numbness, fistula formation, and long-term recurrence
Early Endoscopic Era – ESS (Endoscopic Sinus Surgery)	Endoscopic visualization to improve sinus drainage	Endonasal endoscopic access with limited tissue removal	Improved visualization compared to external approaches; reduced facial morbidity	Limited understanding of mucosal physiology; variable outcomes; less standardized techniques
Functional Endoscopic Sinus Surgery (FESS)	Restoration of physiological sinus ventilation and drainage through osteomeatal complex	Targeted endoscopic intervention (uncinectomy, ethmoidectomy, middle meatal antrostomy)	Mucosal preservation; improved long-term outcomes; standardized technique; reduced complications compared to external surgery	Risk of orbital or skull base injury; outcomes dependent on disease extent and surgical expertise
Image-Guided FESS	Enhanced anatomical precision using real-time navigation	FESS combined with CT-based navigation systems	Improved safety in complex anatomy and revision cases; greater surgical confidence	Increased cost; dependence on technology; limited added value in uncomplicated cases
Balloon Sinuplasty (BSP)	Ostial dilation without tissue or bone removal	Catheter-based balloon dilation of sinus ostia	Minimally invasive; preservation of mucosa; reduced bleeding, operative time, and postoperative care	Limited efficacy in advanced disease, nasal polyposis, osteitis, or extensive inflammation
Thinvasive Surgery (Contemporary Concept)	Personalized, function-preserving modulation of key endonasal structures	Selective use of balloon dilation, limited FESS, and adjunctive technologies (e.g., QMR, KOS)	Maximizes mucosal preservation; individualized treatment; reduced surgical trauma; faster recovery	Limited high-level evidence; lack of standardized protocols; requires careful patient selection

Initial management focuses on evidence-based medical therapy, including intranasal corticosteroids, nasal irrigation, and, when indicated, biologic agents. Functional assessment using nasal endoscopy, validated symptom scoring systems, such as SNOT-22, and adjunctive modalities like QMR and Ozilia-KOS complement radiologic findings in evaluating treatment response and stratifying patients for either continued conservative management or surgical consideration [56,57]. This stepwise strategy aims to avoid overtreatment while ensuring timely intervention in appropriately selected patients. As emphasized in recent literature, structured use of both functional and anatomical assessments facilitates more precise decision-making and preserves mucosal integrity [12].

**Figure 3:**
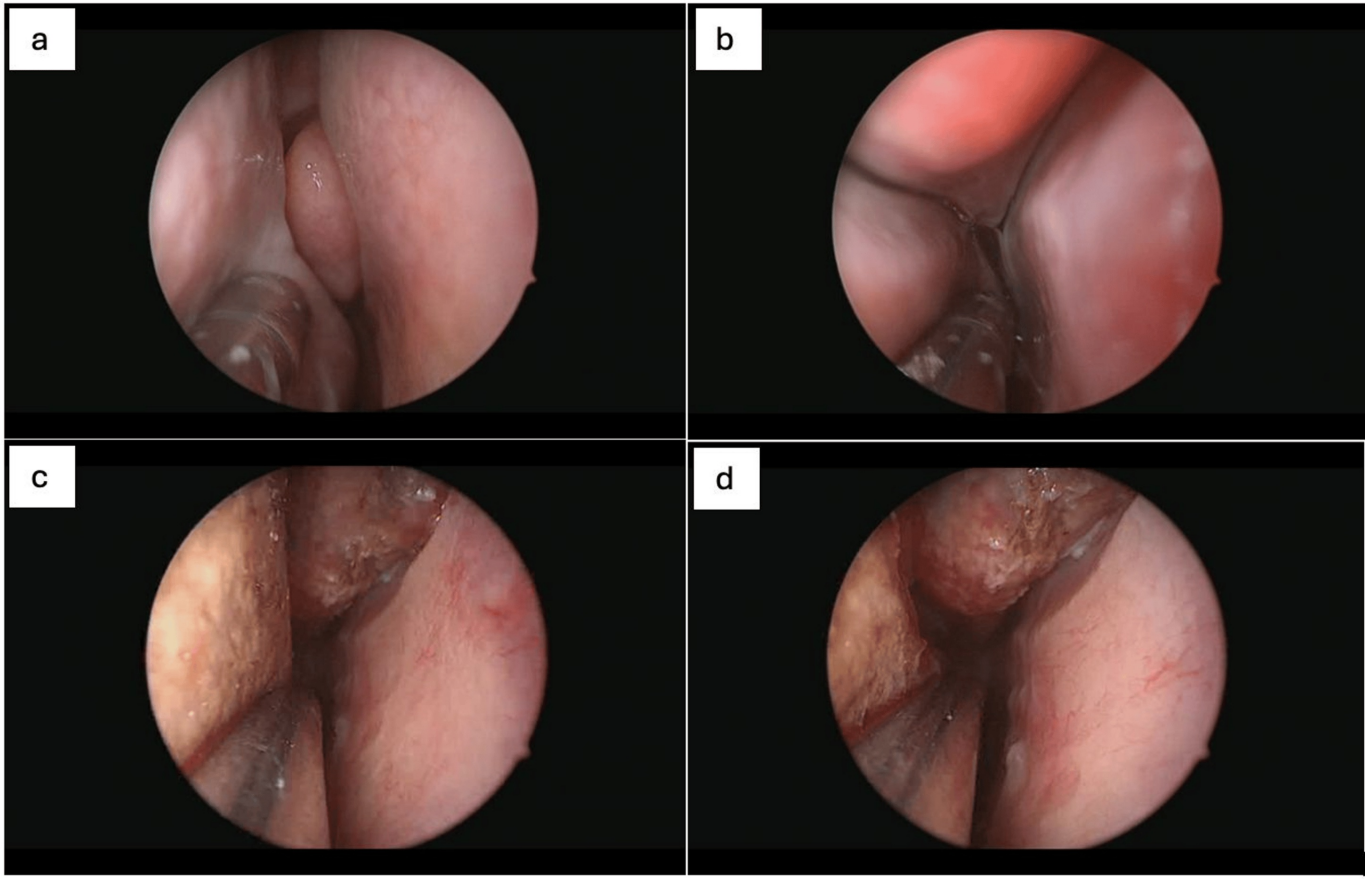
Preoperative view of the anterior aspect of the right inferior and medial meatus with visible turbinate hypertrophy (a). Posterior aspect before intervention, showing narrow anatomical configuration and contact between structures (b). Postoperative image of the same anterior region showing significant widening of the nasal airway and decompressed turbinates (c). Posterior view after combined balloon sinuplasty and turbinoplasty, revealing expanded meatal space with preserved mucosa (d). Consent from the patient to submit images has been obtained prior to submission to the journal.

The Thinvasive approach exemplifies this philosophy by incorporating minimally invasive technologies, such as QMR and Ozilia-KOS, alongside established techniques like BSP and FESS (Figure 3). These modalities are applied selectively based on objective findings and patient preferences, in line with shared decision-making models [1]. Ultimately, this framework supports the overarching goal of function-preserving, precision-driven endonasal sinus surgery.

Limitations

Evidence supporting the Thinvasive approach, QMR, and Ozilia-KOS remains limited and largely derived from small-scale or uncontrolled studies. More rigorous research, including randomized controlled trials in the CRS population, is necessary to determine the therapeutic efficacy, duration of benefit, and appropriate patient selection criteria for this approach.

## Conclusions

As technology advances and our understanding of CRS pathophysiology deepens, the future of sinus surgery is unlikely to be defined by a single dominant technique. Instead, it will evolve into a continuum of tailored approaches. Surgeons are increasingly expected to move beyond procedural expertise toward strategic decision-making, choosing tools and methods based not only on anatomical factors but also on mucosal biology, patient comorbidities, and individual treatment goals. FESS remains an essential tool, particularly for managing advanced disease, nasal polyposis, and revision cases. BSP has proven highly effective for well-selected patients with non-polypoid CRS and favorable anatomy, offering the benefits of reduced surgical trauma and faster recovery. Thinvasive multimodal approaches, blending balloon technology, QMR, Ozilia-KOS, and personalized selection frameworks, present a hybrid model that allows clinicians to achieve therapeutic goals while preserving mucosal integrity and targets the underlying causes of inflammation and disease, rather than addressing only the consequences.
